# The impact of substrate stiffness on morphological, transcriptional and functional aspects in RPE

**DOI:** 10.1038/s41598-024-56661-7

**Published:** 2024-03-29

**Authors:** Lasse Wolfram, Clara Gimpel, Melanie Schwämmle, Simon J. Clark, Daniel Böhringer, Günther Schlunck

**Affiliations:** 1 Eye Center, Medical Center, Faculty of Medicine, University of Freiburg, Freiburg, Germany; 2 Department for Ophthalmology, Institute for Ophthalmic Research, Eberhard Karls University of Tübingen, Tübingen, Germany; 3 Department for Ophthalmology, University Eye Clinic, Eberhard Karls University of Tübingen, Tübingen, Germany; 4 Department of Neurology, Schlosspark-Klinik Charlottenburg, Berlin, Germany; 5 Faculty of Biology, University of Freiburg, Freiburg, Germany; 6 Lydia Becker Institute of Immunology and Inflammation, University of Manchester, Manchester, UK

**Keywords:** miRNA, ECM, Substrate stiffness, Extracellular matrix, Next-generation sequencing, Macular degeneration

## Abstract

Alterations in the structure and composition of Bruch’s membrane (BrM) and loss of retinal pigment epithelial (RPE) cells are associated with various ocular diseases, notably age-related macular degeneration (AMD) as well as several inherited retinal diseases (IRDs). We explored the influence of stiffness as a major BrM characteristic on the RPE transcriptome and morphology. ARPE-19 cells were plated on soft ($E={30}\,\hbox {kPa}$) or stiff ($E={80}\,\hbox {kPa}$) polyacrylamide gels (PA gels) or standard tissue culture plastic (TCP). Next-generation sequencing (NGS) data on differentially expressed small RNAs (sRNAs) and messenger RNAs (mRNAs) were validated by qPCR, immunofluorescence or western blotting. The microRNA (miRNA) fraction of sRNAs grew with substrate stiffness and distinct miRNAs such as miR-204 or miR-222 were differentially expressed. mRNA targets of differentially expressed miRNAs were stably expressed, suggesting a homeostatic effect of miRNAs. mRNA transcription patterns were substrate stiffness-dependent, including components of Wnt/beta-catenin signaling, Microphthalmia-Associated Transcription Factor (MITF) and Dicer. These findings highlight the relevance of mechanical properties of the extracellular matrix (ECM) in cell culture experiments, especially those focusing on ECM-related diseases, such as AMD.

## Introduction

AMD is a chronic progressive disease which leads to degeneration of the central retina and is the leading cause of incurable blindness worldwide in the elderly^1^. As a multifactorial disease it is driven by a complex interplay of genetic risk variants, natural aging and lifestyle factors, such as smoking status and nutritional intake^2,3^. Based on clinical examination and histopathological findings, AMD can be graded as an early, intermediate or late stage of disease. Drusen, lipid-rich extracellular deposits between the RPE and BrM^4^, and pigment irregularities are signs of early and intermediate AMD whereas geographic atrophy (GA) and choroidal neovascularization (CNV) define late stages of disease^5,6^. Despite intense investigational efforts in the field of AMD and the approval of Pegcetacoplan in early 2023 as the first therapy for late-stage dry AMD^7^, the knowledge on the exact pathophysiology still remains incomplete and we still lack effective treatments for earlier stages of AMD that have the potential to prevent irreversible visual impairment altogether, whereas anti-VEGF therapy is available for the exudative form of AMD^8,9^.

Cells perceive the mechanical properties of their environment, the ECM, and are directly influenced by its changes^10^. They are equipped with a number of cell adhesion proteins that enable cell-cell and cell-matrix interaction, including integrins, cadherins, Ig-CAMs and selectins^11^. Contractility^12^ and migration^13^ as well as morphology^14^, proliferation^15^, differentiation^16^ and apoptosis^17^ of cells are affected by changes in the biomechanical properties of the ECM. Furthermore, RPE cells show higher resilience to oxidative stress when grown on ECM components^18^. Stiffness-dependent changes in gene expression patterns and growth factor signaling have been described in cells of the eye^19,20^. These effects depend on the individual cell type and the adhesion receptor profile involved^21^ and have yet not been comprehensively described for RPE cells.

The loss of function of elastic connective tissue components and an increasing tissue stiffness play a decisive role in the development of common age-associated diseases such as atherosclerosis^22^. In ocular diseases such as AMD and primary open-angle glaucoma, changes in tissue stiffness have also been observed^23,24^, although the pathophysiological significance of these changes is not yet fully understood. Also, the deposition of extracellular material as linear and laminar deposits or drusen in the region of BrM is very likely to be accompanied by changes in the biomechanical properties of the surrounding tissue. Indeed, increased stiffness of the BrM, likely due to structural changes occurring in BrM with age, has previously been observed in tissues of advanced age^25,26^.

A measurement of the Young’s modulus of lipofuscin-containing basal deposits or drusen is not available at the time of writing. However, for most human tissues, a range of stiffness between $E={1}\,\hbox {kPa}$ and $E={80}\,\hbox {kPa}$ can be assumed^16^. In contrast, the stiffness of TCP conventionally used in cell culture is estimated to be $E_{\text {TCP}}\approx {3}\,\hbox {GPa}\approx$ 3,000,00$0 \,\hbox {kPa}$^27^. Commercially available porous polyethylene terephthalate (PET) Transwell membranes display a Young’s modulus of about $E_{\text {TW}}\approx {2}\,\hbox {GPa}\approx$ 2,000,00$0 \,\hbox {kPa}$^28,29^ and are therefore only slightly softer than TCP. Both Young’s moduli are several orders of magnitude higher than the range of stiffness measured in most human tissues (about $E={1}\,\hbox {kPa}$ to $E={80}\,\hbox {kPa}$) and the substrates compared here ($E={30}\,\hbox {kPa}$ and $E={80}\,\hbox {kPa}$). Recent investigations on the impact of substrate stiffness on RPE cells already revealed valuable findings, but the state of confluence of RPE as a type of epithelial cells^30^ has not been particularly considered in this purpose yet^31,32^.

miRNAs are small (approximately $22 \,\hbox {nt}$) non-coding RNAs that suppressively modulate translation and constitute an essential, as yet poorly characterized regulation of protein expression^33–35^. Specific mRNA transcripts can be the target of different miRNAs. At the same time, certain miRNAs bind to response elements of different mRNAs^36^. Thus, miRNA signaling networks can cause complex changes in the transcriptome^37,38^.

In both vascular endothelium and in cartilage cells, modulation of miRNA expression by mechanical stimuli has been described^39,40^. Functionally, finely regulated feedback mechanisms between miRNAs and ECM, such as the regulation of Dicer by MITF may play a central role^41,42^. By modulation of synthesis and turnover of adhesion molecules and their receptors, such as cadherins, integrins and other non-integrin ECM receptors, miRNAs regulate the composition of the ECM^43^. In this way, miRNAs can counteract fluctuations in protein expression to achieve more constant tissue properties to ensure mechanical homeostasis^44^. In the context of age-related tissue changes, possible changes in miRNA expression patterns due to mechanical influences may be of pathophysiological significance.

miRNAs are involved in signaling networks in various different ways. They serve either as an additional layer of transcriptional control or as feed-forward or feedback mediators. Thus, expression within the cell can be finely regulated and its level stabilized. Also in the context of Wnt signaling pathways, the majority of mediators are regulated by miRNAs^45,46^, thus a close reciprocal interaction can be assumed^47^. Distinct factors and target proteins of the Wnt/beta-catenin signaling pathway with a known impact on RPE were specifically further investigated in this work in order to describe stiffness-dependent, likely miRNA-related expression patterns.

Wnt ligands show an ECM stiffness-dependent expression^48^ and are essential for the development and maintenance of homeostasis in nearly all tissue types^49^. Since differentiation and pigmentation are interrelated in RPE, regulation by a common signaling pathway, e.g. the Wnt/beta-catenin pathway, is being discussed^50^. Free beta-catenin serves as a central signaling molecule for both Wnt/beta-catenin signaling and cadherin-mediated cell adhesion, suggesting a potentially relevant convergence and competition of these pathways^51,52^. Due to mechanosensitive regulation of cadherin-mediated intercellular junctions^53,54^, the competition between Wnt-associated and cadherin-associated functions of free beta-catenin may become even more relevant with increasing tissue stiffness and number of cell-cell-junctions.

Activation of the Wnt/beta-catenin signaling pathway plays a pathogenic role in AMD^55^. MITF promotes differentiation of RPE cells^56^ and serves both as a downstream target and a nuclear mediator of Wnt ligands^57^. Tyrosinase Related Protein 1 (TYRP1) is a melanocyte-specific gene product essential for melanin synthesis^58^. As a target gene of the transcription factor MITF, its expression is subject to direct regulation by MITF^59^. Dicer, as a central element for the processing of miRNAs^60^, shows direct transcriptional targeting by MITF^41,42^.

## Results

### ECM stiffness alters morphology and RNA profile of RPE cells

To directly address the question whether or not substrate stiffness altered RPE cell morphology, ARPE-19 cells were cultivated on different substrates. On PA gels of higher stiffness, RPE attained a characteristic polygonal epithelial morphology, whereas on softer PA gels a less uniformed cell lawn with a broader range of cell sizes and blank spaces was observed. These blank spaces were closed only after approximately one week in culture, resulting in a more heterogeneous morphology. A comparison of the morphology of ARPE-19 after three weeks of culturing on the different substrates is shown in Supplementary Fig. S1.

**Figure 1:**
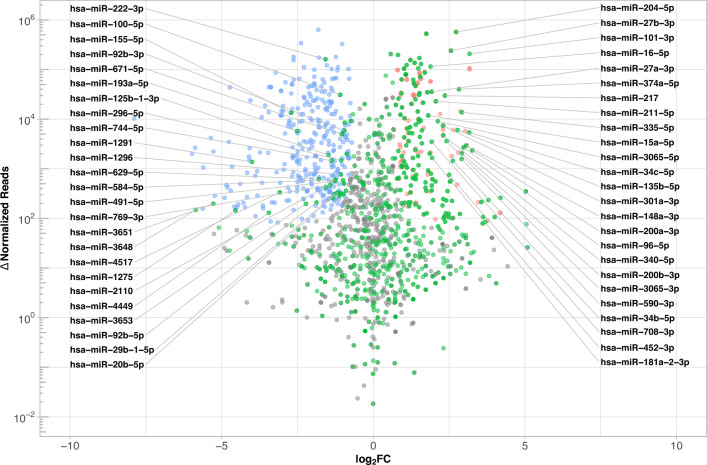
Differential expression of sRNAs in ARPE-19 cells (NGS). Comparison of the groups $E={30}\,\hbox {kPa}$ and $E={80}\,\hbox {kPa}$, visualization as MD-plot. Relative difference in expression as $\log _2 FC$ is plotted against absolute difference of normalized reads. sRNAs with a probability of differential expression $q\ge {0.8}$ are colored in blue or in red (597), differentiation between sRNAs with lower expression on stiffer substrates compared to softer substrates (354, 35 miRNAs amongst them, blue) and vice versa (243, 135 miRNAs amongst them, red). miRNA fraction emphasized (green), declaration of 25 miRNAs each with highest significance for differential expression.

**Figure 2:**
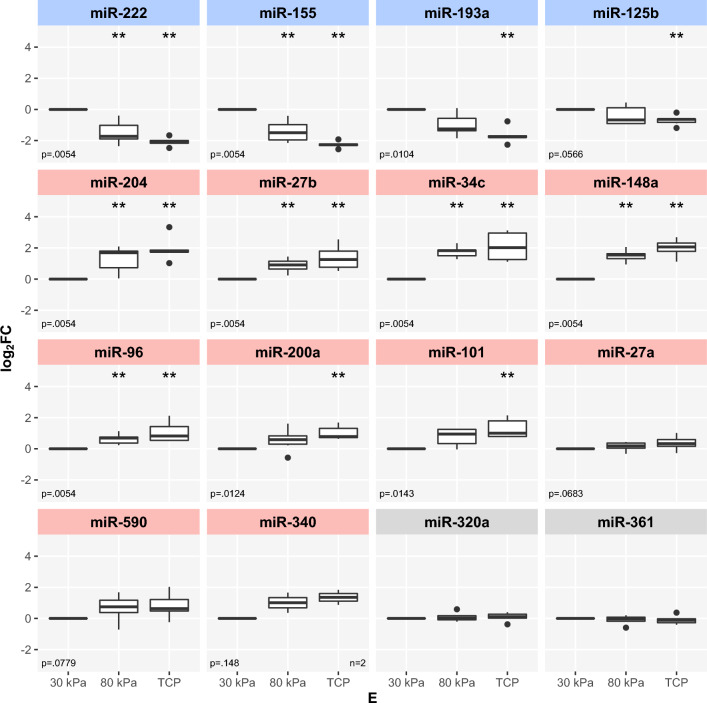
Stiffness-dependent expression of miRNAs in ARPE-19 cells (qPCR). Expression data from $n={6}$ independent experiments ($n={2}$ for miR-340), visualization as $\log _2 FC$ with $E={30}\,\hbox {kPa}$ as reference. Coloring of groups “decrease in expression with higher substrate stiffness” (blue) and “increase in expression with higher substrate stiffness” (red), based on NGS-data (Fig. 1). Normalization on *hsa-miR-320a* and *hsa-miR-361-5p* as endogenous controls (gray). Statistical significance between each two interventions was assessed by Wilcoxon-Mann-Whitney test with $E={30}\,\hbox {kPa}$ as reference. $p\le 0,05$ (*), $p\le 0,01$ (**) and $p\le 0,001$ (***). Global p-values were determined using Kruskal-Wallis test. Benjamini-Hochberg procedure was applied for multiple testing correction.

The stiffness of the substrates used in these experiments also changed the expression levels of miRNAs in the RPE cells. NGS of sRNAs revealed transcription of 2019 individual genes, amongst them 755 different miRNA genes, 569 of these on all substrates. The majority of differentially expressed sRNAs were more highly expressed on softer substrates (354 out of 597 $\hat{=}$ 59,30%). However, a relatively low proportion of miRNAs was found within this group (35 out of 354 $\hat{=}$ 9,89%). The proportion of miRNAs within the group of differentially expressed sRNAs with higher expression on stiff substrates (243 out of 597 $\hat{=}$ 40,70%) was significantly higher (135 out of 243 $\hat{=}$ 55${\overline{5}}\%$) (Fig. 1). sRNA NGS data was further validated by qPCR and found to be highly consistent for differentially expressed miRNAs (Fig. 2).

**Figure 3:**
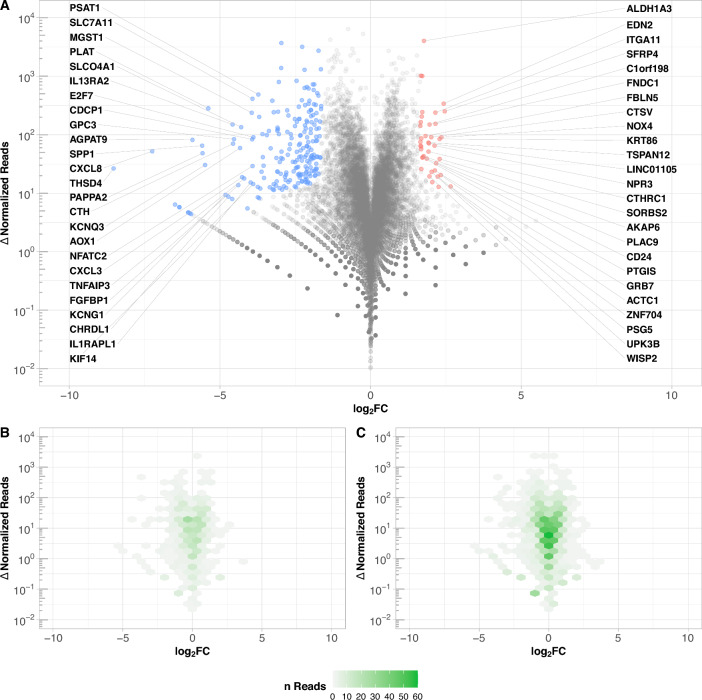
Differential expression of mRNAs and distribution of predicted miRNA targets in ARPE-19 cells (NGS). Relative difference in expression as $\log _2 FC$ is plotted against absolute difference of normalized reads. (**A**) mRNAs with a probability of differential expression $q\ge {0.8}$ are colored (232), differentiation between lower expressed mRNAs on stiffer substrates compared to softer substrates (195, blue) and vice versa (37, red). Declaration of 25 mRNAs each with highest significance for differential expression. (**B**, **C**) Distribution of the predicted targets of the colored 35 miRNAs with lower expression on stiffer substrates compared to softer substrates (B, 668) and the colored 135 miRNAs with higher expression on stiffer substrates compared to softer substrates (C, 1696) in Fig. 1, based on a $P_{\text {CT}}>{0.9}$. Color intensity (green) based on the density of summarized dots.

Additionally, the NGS analysis detected transcripts of 20,045 individual genes. A visualization of the differential expression analysis, as well as the distribution of predicted targets of differentially expressed miRNAs, is shown in Fig. 3. Interestingly, based on a $P_{\text {CT}}>{0.9}$ a huge overlap between the target sequences of miRNAs with lower and higher expression on stiff substrates compared to soft substrates can be seen.

### In silico pathway analysis of NGS data

**Figure 4:**
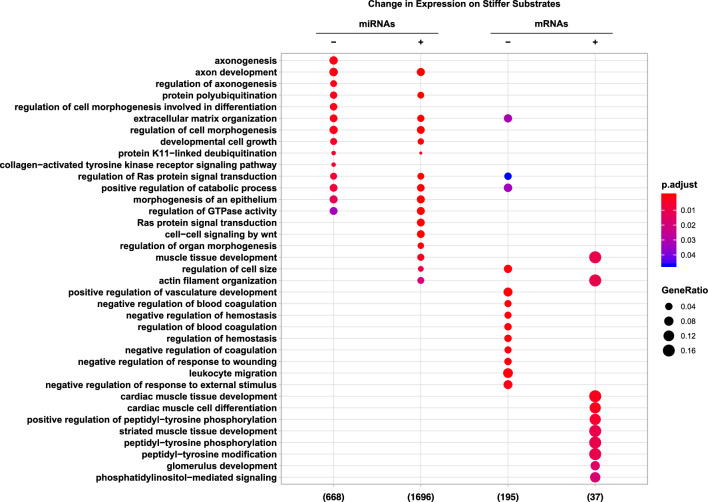
Comparative visualization of *in silico* *GO*-enrichment analyses (NGS). Classification based on NGS data: Pathways affected by the targets of miRNAs with lower expression on stiffer substrates (miRNAs-) and miRNAs with higher expression on stiffer substrates (miRNAs+) as well as pathways affected by the mRNAs with lower expression on stiffer substrates (mRNAs-) and mRNAs with higher expression on stiffer substrates (mRNAs+) in ARPE-19 cells. Declaration of the number of underlying genes. Color-coded adjusted *p*-value, size-coded GeneRatio.

A comparative visualization of *in silico* *Gene Ontology (GO)* enrichment analyses based on the particular NGS datasets is shown in Fig. 4. Based on the mRNA datasets, no overlap of *GO* terms was detected. The pathways of differentially more lowly and more highly expressed mRNAs seem mutually exclusive. mRNAs showing lower expression on stiffer substrates are predominantly belonging to angiogenesis-related pathways, whereas more highly expressed mRNAs are mostly associated with myofilament development and muscular tissue assembly.

On the contrary, miRNAs differentially expressed in a stiffness-dependent manner targeted primarily mRNAs whose expression levels were unaffected by ECM stiffness. In line with this, an extensive overlap of functional *GO* term families was shown within miRNA targets. Commonly regulated pathways include ECM organization and regulation of cellular differentiation and morphology, Ras-mediated differentiation and proliferation as well as axon development and regulation. As an exception, mRNAs involved in certain pathways, e.g. “cell-cell signaling by Wnt”, are most exclusively targeted by miRNAs more highly expressed on stiffer substrates.

### Stiffness-dependent changes in Wnt/beta-catenin signaling

Based on our data suggesting the regulation of Wnt-mediated signaling by mechanosensitive miRNAs in ARPE-19, we further investigated stiffness-related changes in the expression of factors and targets of Wnt/beta-catenin signaling with the help of the RT^2^ Profiler PCR Array. Out of 84 investigated genes, 17 showed a stiffness-dependent expression (Supplementary Table 1). Additional expression analyses of agonists and antagonists of Wnt/beta-catenin signaling by qPCR are shown in Supplementary Fig. S2.

**Figure 5:**
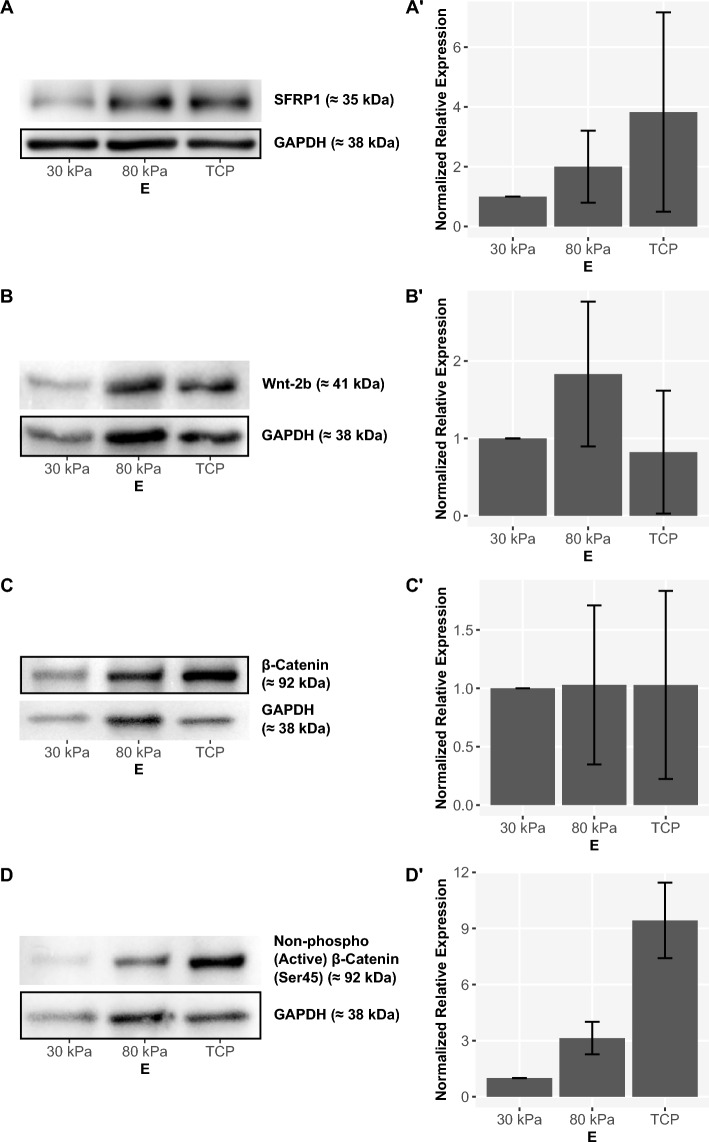
Stiffness-dependent expression of different members of the Wnt/beta-catenin signaling pathway in ARPE-19 cells (western blot). Example blot (left side) and semiquantitative analysis with visualization of normalized expression based on GAPDH as endogenous control and error bars depicting standard deviation (right side). (**A**) SFRP1, $n={4}$; (**B**) Wnt-2b, $n={3}$; (**C**) beta-catenin, $n={3}$; (**D**) Non-phospho (active) beta-catenin (Ser45), $n={3}$. The full western blots are available in Supplementary Figs. S3–S9.

Subsequent analysis of specific agonists and antagonists of Wnt/beta-catenin signaling at the protein level, using western blotting, is shown in Fig. 5. Secreted Frizzled Related Protein 1 (SFRP1), a potent antagonist of Wnt/beta-catenin signaling with impact on cell differentiation and cell proliferation^61–64^, showed a tendency to higher expression levels on stiffer substrates (Fig. 5A). These changes are concordant to the stiffness-dependent changes in expression of *SFRP1* detected by qPCR (Supplementary Fig. S2, SFRP1). Wnt-2b, a direct agonist of Wnt/beta-catenin signaling, also showed higher expression levels on stiffer substrates compared to softer substrates (Fig. 5B) and therefore a similar trend compared to our findings in qPCR analyses (Supplementary Fig. S2, WNT2B). After expression analyses of single factors of Wnt/beta-catenin signaling, we further investigated the stiffness-dependent pathway activity. We analyzed the expression of the decisive protein beta-catenin and its active, non-phosphorylated version, non-phospho (active) beta-catenin (Ser45). Whereas we barely see any stiffness-dependent effect on the expression of beta-catenin (Fig. 5C), a significant increase of the isolated active version of the protein on stiffer substrates was detected (Fig. 5D).

### Stiffness-dependent expression of MITF and Dicer

**Figure 6:**
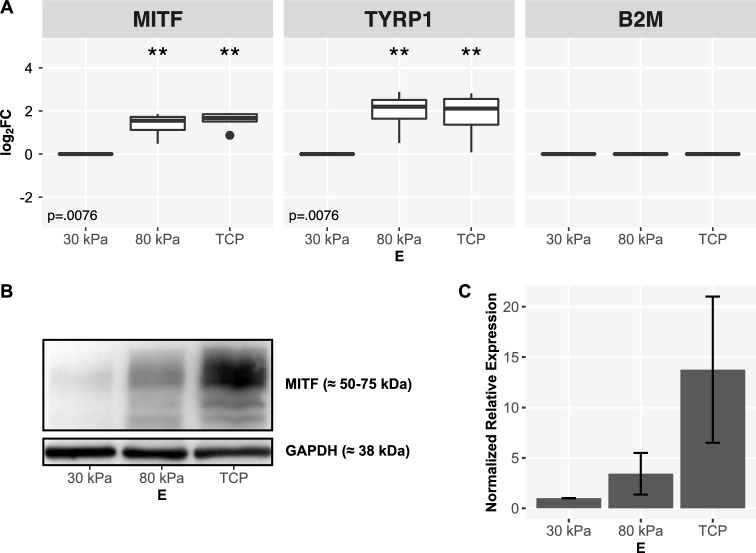
(**A**) Stiffness-dependent expression of *MITF* and *TYRP1* in ARPE-19 cells (qPCR). Expression data from $n={5}$ independent experiments, visualization as $\log _2 FC$ with $E={30}\,\hbox {kPa}$ as reference. Normalization on *B2M* as endogenous control. Statistical significance between each two interventions was assessed by Wilcoxon-Mann-Whitney test with $E={30}\,\hbox {kPa}$ as reference. $p\le 0,05$ (*), $p\le 0,01$ (**) and $p\le 0,001$ (***). Global p-values were determined using Kruskal-Wallis test. Benjamini-Hochberg procedure was applied for multiple testing correction. (**B, C**) Stiffness-dependent expression of MITF in ARPE-19 cells (western blot). Example blot (**B**) and semiquantitative analysis from $n={4}$ independent experiments with visualization of normalized expression based on GAPDH as endogenous control and error bars depicting standard deviation (**C**). The full western blots are available in Supplementary Figs. S10, S11.

MITF is a transcription factor and a known downstream target of Wnt/beta-catenin signaling with a defined impact on RPE differentiation^56^ and was therefore identified for further analysis in our study. MITF showed a significant increase in expression on stiffer substrates both in terms of gene transcription and protein expression (Fig. 6). This result is supported by the increased expression of *TYRP1*, a target gene of MITF^59^, in RPE cells grown on stiffer substrates (Fig. 6A). These findings indicate not only an increase of expression but also activity of the pivotal transcription factor MITF in ARPE-19 when cultivated on stiffer substrates.

**Figure 7:**
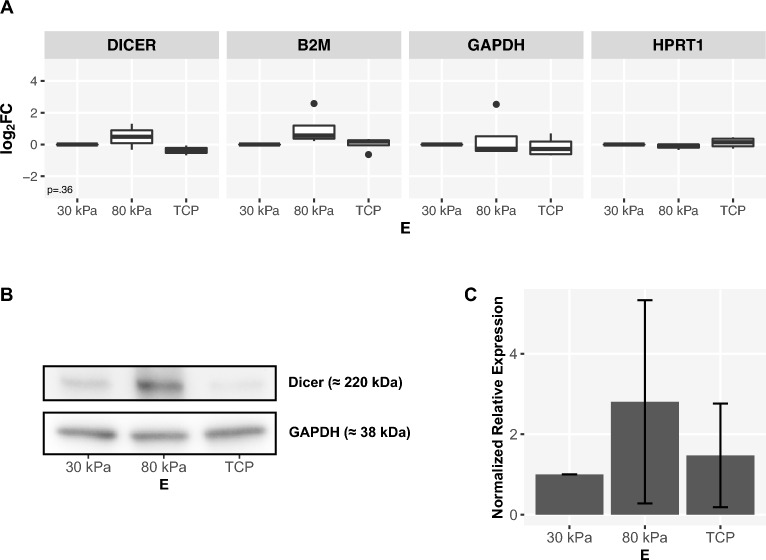
(**A**) Stiffness-dependent expression of *DICER* in ARPE-19 cells (qPCR). Expression data from $n={4}$ independent experiments, visualization as $\log _2 FC$ with $E={30}\,\hbox {kPa}$ as reference. Normalization on *B2M*, *GAPDH* and *HRPT1* as endogenous controls. Statistical significance between each two interventions was assessed by Wilcoxon-Mann-Whitney test with $E={30}\,\hbox {kPa}$ as reference. $p\le 0,05$ (*), $p\le 0,01$ (**) and $p\le 0,001$ (***). Global p-values were determined using Kruskal-Wallis test. Benjamini-Hochberg procedure was applied for multiple testing correction. (**B, C**) Stiffness-dependent expression of Dicer in ARPE-19 cells (western blot). Example blot (**B**) and semiquantitative analysis from $n={3}$ independent experiments with visualization of normalized expression based on *GAPDH* as endogenous control and error bars depicting standard deviation (**C**). The full western blots are available in Supplementary Figs. S12, S13.

The endoribonuclease Dicer, recently reported as being directly transcriptionally targeted by MITF^41,42^, plays a critical role in the processing of miRNAs^60^. To address the question of a possible Wnt-mediated stiffness-dependent change in the expression of Dicer, an investigation was first performed by qPCR (Fig. 7A). No significant changes were detected in the expression of mRNA of Dicer between the studied substrates. In contrast to mRNA expression, protein expression of Dicer showed a tendency to higher expression levels on stiffer substrates compared to softer substrates in western blot (Fig. 7B and C).

## Discussion

On stiff substrates, ARPE-19 cells showed a nearly homogeneous growth pattern of small cells in a cobblestone-like conformation, which attained increasing heterogeneity and cell size variation with decreasing substrate stiffness (Supplementary Fig. S1, ${30}\,\hbox {kPa}$). This is reminiscent of reports on RPE flat mount specimens retrieved from AMD eyes, which revealed extensive cytoskeletal changes including a loss of regular polygonal geometry and cells of different sizes and shapes in AMD lesions^65^. Depending on the size and nature of RPE defects, different repair mechanisms and subsequent RPE growth patterns have been postulated. The emergence of large, multinucleated cells has been discussed to occur in the course of hypertrophic restoration of a confluent cell layer after cell loss in AMD lesions^66^.

The present study indicates an impact of ECM stiffness on RPE cell adhesion, cytoskeleton, cell morphology and epithelial differentiation. This is in line with earlier reports on the role of mechanotransduction in the formation of focal complexes and focal contacts^67^ and its impact on cell adhesion and signaling^19,22^. Changes in cell adhesion were also observed in RPE cell layers of AMD patients *ex vivo*^68^. In experiments using human donor preparations with submacular drusen, it was noted that RPE cells were “loosely attached and could be peeled off as a sheet”^68^. The total stiffness of BrM has been studied in bulk measurements and an age-dependent stiffness increase was reported^25^, but possible changes in adhesive properties and the stiffness of basal deposits have not been characterized. A loss of adhesion of RPE in the area of lipofuscin-containing basal deposits and drusen in the course of AMD is conceivable and may have a role in AMD pathogenesis as a possible driver of disease.

ECM characteristics such as substrate stiffness, microstructural properties or adhesion ligand densities are frequently not addressed in *in vitro* experiments^69^. In light of the significant differences in the composition and properties of tissue culture plastic and human BrM, results of standard cell culture experiments need to be interpreted with caution. In general, biomechanical conditions have multiple cell adhesion-mediated effects on the transcriptome^16,21^. Few reports addressed the influence of substrate rigidity on human RPE cells and revealed decreased phagocytosis capacities^31^ and increased expression of inflammatory markers^32^ with increasing substrate stiffness. Our work focussed on analyzing the impact of substrate stiffness in a physiologically relevant stiffness range^16^ and especially considered the importance of confluency for RPE as an epithelial cell type^30^. It was our goal to explore rigidity-dependent changes in the transcription of mRNAs as well as small RNAs and to characterize possible regulatory interactions. Small RNAs comprise miRNAs, small nucleolar RNAs (snoRNAs) with a reported detection in murine retinal tissues^70^, piwi-interacting RNAs (piRNAs) with supposed gene regulatory function in the germ line^71,72^ and others. Our data suggest that sRNA transcription is strongly influenced by ECM stiffness, whereas not homogeneously distributed across RNA classes. sRNA subtype distribution is shifted towards a higher relative abundance of miRNAs on stiff PA gels, whereas piRNAs and snoRNAs were the most abundant subtypes on soft PA gels. Regarding the observed 597 differentially expressed sRNA transcripts, a majority (354) was more strongly expressed on soft PA gels as compared to 243 sRNAs on stiff substrates. Due to a comparatively high proportion of miRNAs within the group of differentially expressed sRNAs with higher abundance on stiffer substrates, an overall increased expression of the miRNA fraction has been detected on stiffer substrates.

A number of differentially expressed miRNAs are assumed to be of particular importance in RPE. Among the miRNAs with the highest significance for differential expression (Fig. 1), miR-200a/200b, miR-204/211 and miR-222 have been described as being enriched in fetal human RPE by 10- to 754-fold compared with neuroretina or choroid^73^. MiR-204, a miRNA well studied in RPE, has been associated with the maintenance of epithelial morphology and physiology^56,73–75^. The reduced expression of differentiation-related miRNAs on softer substrates is consistent with the observed morphology of the cells (Supplementary Fig. S1).

Due to an increase in incidence and prevalence of AMD disease and a progress in its therapeutic options, even for dry AMD (Pegcetacoplan^7^), several studies focusing on miRNAs as potential diagnostic and prognostic biomarkers of AMD have been published^76,77^. However, comparability between miRNA abundance in peripheral blood samples and ocular samples has increasingly been questioned^78^. As we investigated miRNA levels in RPE cells, a comparison to studies of ocular samples seems most appropriate. Upregulation of miR-125b and miR-155 in AMD-affected macular region of retinal tissues revealed high consistency with upregulation of these miRNAs in ARPE-19 cells grown on softer substrates (Fig. 2)^76,79^. Furthermore, downregulation of miR-200a in aqueous samples of AMD patients, possibly reflecting the mechanisms of angiogenesis in AMD, was consistent with upregulation of miR-200a in ARPE-19 on stiffer substrates (Fig. 2)^78^. Taken together, ARPE-19 cells grown on softer substrates ($E={30}\,\hbox {kPa}$) do not only appear to be more AMD like in structure, but also, based on a limited number of studies investigating miRNA abundance in ocular samples from AMD patients, seem to show a more comparable miRNA expression profile to ocular samples from AMD patients. In contrast, ARPE-19 cells grown on stiffer substrates ($E={80}\,\hbox {kPa}$) appear more comparable to physiological human RPE.

Contrary to expectations, target prediction analyses revealed that the most significant changes in mRNA expression induced by modification of tissue stiffness are not related to differences in miRNA expression profiles. Predicted miRNA target sequences showed a predominantly central distribution in mRNA NGS data (Fig. 3). It is therefore likely that miRNAs exert a more fine-regulatory effect on the modification of the biomechanical nature of the environment. This finding is highly consistent with a previously described homeostatic function of miRNAs to buffer fluctuations in protein levels caused by changes in transcriptional inputs or extracellular factors^44^. A recently postulated regulation of not only expression but also expression variability of target genes by miRNAs^80^ is coherent with our findings of miRNA targets predominantly showing only minor stiffness-dependent expression changes. A contribution to stabilizing cellular homeostasis and maintaining cellular integrity and stability by the shown overall increased expression of miRNAs on stiffer substrates is conceivable.

Whereas *GO* term families (Fig. 4) of up- or downregulated mRNAs did not show substantial intersection, differentially expressed miRNAs targeted mRNAs of similar functional entities, suggesting a miRNA-based regulation of similar *GO* term families in both softer and stiffer tissue environments. These findings are consistent with a previously described functional redundancy of miRNAs^81^. Only little overlap can be seen when comparing *GO* term families of differentially expressed mRNAs and targets of differentially expressed miRNAs. These observations suggest that stiffness-dependent changes in mRNA transcription were not the result of alterations in the miRNA expression pattern. Moreover, expression levels of mRNAs targeted by differentially expressed miRNAs tended to be unaffected by changes in substrate stiffness. These findings strongly support the notion that mechanosensitive miRNAs serve to stabilize mRNA expression patterns as has also been reported in vascular endothelial cells^44^.

Interestingly, mRNAs associated to the GO term “cell-cell signaling by wnt” were targeted only by miRNAs upregulated on stiff PA gels. Wnt signaling has an important role in RPE differentiation^50^ and a stiffness-dependent miRNA-mediated change in the activity of the Wnt/beta-catenin signaling pathway can be suspected, which may be important for stiffness-dependent differentiation of RPE.

The present work demonstrates a stiffness-dependent expression of a number of miRNAs related to Wnt/beta-catenin signaling in RPE^45–47^, such as activation of miR-34c expression by Wnt/beta-catenin signaling^82^, regulation of the Wnt antagonist SFRP1 by miR-27a and miR-27b^83^ and suppression of Wnt/beta-catenin signaling by direct interaction of miR-200a with beta-catenin^84,85^. miRNAs crosstalk with a variety of the key cellular signaling networks such as Wnt to control stem cell activity in maintaining tissue homeostasis^86^.

Active beta-catenin showed a significant increase in expression on stiffer substrates (Fig. 5), suggesting an overall higher activity of the Wnt/beta-catenin signaling pathway on these substrates. When analyzing stiffness-dependent expression changes of antagonists and agonists of Wnt/beta-catenin signaling, parallel tendencies can be detected. This finding is consistent with negative feedback mechanisms leading to a complex, finely tuned regulation within the pathway^47^. In total, an increase in the activity of the Wnt/beta-catenin signaling pathway on stiffer substrates can be seen. This cannot be explained exclusively by the increased expression of beta-catenin, but rather based on an actual increase in activity of beta-catenin due to decreased phosphorylation at Ser45 and consecutive decreased degradation of beta-catenin.

A number of differentially expressed miRNAs are known to be associated with downstream targets of Wnt-signaling. For instance, miR-148a and miR-101 show a significant impact on MITF expression in melanoma cells^87^. MITF, in turn, regulates miR-204/miR-211 expression and promotes epithelial differentiation in RPE^56^. Both qPCR and western blotting revealed a significant increase in the expression and activity of MITF on stiffer substrates. Increased mRNA expression of *TYRP1* indicates not only higher expression but also increased activity of MITF as a transcription factor on stiffer substrates^59^. Significant expression changes of miR-204 and MITF in line with morphological changes of ARPE-19 in a stiffness-dependent manner highlights the relevance of biomechanical properties in RPE.

Dicer showed a tendency of increased protein expression on stiffer substrates, however no significant changes on transcriptional level were detectable when examined by qPCR (Fig. 7). This could be due to different turnover rates or a possible rapid degradation of its mRNA and therefore consistent with Dicer being subject to multiple layers of regulation^88^. MITF triggers the expression of Dicer in melanocytes^41,42^ and could therefore contribute to the observed global enhancement of miRNA expression on stiffer substrates, probably affecting a variety of signaling pathways. Isolated exploration of the impact of the individual proteins discussed here could represent a helpful further step.

Initial experiments were conducted to collect pilot data, with results from NGS analysis of $n={1}$ samples serving as an initial step towards generating hypotheses and identifying relevant miRNAs and mRNAs. Literature research indicated consistent changes in relevant pathways in RPE cells when grown on amniotic membranes ($E={1.22}\,\hbox {kPa}$ to $E={5.50}\,\hbox {kPa}$) compared to TCP coated with matrigel ($E={70.72}\,\hbox {MPa}$ to $E={175.93}\,\hbox {MPa}$)^89^. While stiffness ranges may differ from those in our study and the comparison between Atomic force microscopy (AFM) measurements and nanoindentation may not be straightforward, statements regarding ’soft substrate vs. TCP’ should nonetheless be fundamentally comparable. Consistent with our pathway analysis (Fig. 4), significant changes in pathways related to ECM-receptor interaction, regulation of actin cytoskeleton as well as complement and coagulation cascades are described. Moreover, an increase in the expression of epithelial-mesenchymal transition (EMT) promotion factors like TGFB1 (Gene Expression Omnibus (GEO) repository, https://www.ncbi.nlm.nih.gov/geo/query/acc.cgi?acc=GSE225642) is consistent with the emphasized trend towards an increase in EMT-related transcripts on TCP coated with matrigel when compared with amniotic membranes. All miRNAs and mRNAs deemed relevant for downstream analysis and discussion were subsequently validated by qPCR in $n={6}$ independent experiments for miRNAs and $n={4}$ independent experiments for mRNAs. The comprehensive qPCR-based validation process ensured a robust and reliable foundation for further analyses.

As primary human RPE cells can hardly be passaged in culture, most *in vitro* studies use immortalized RPE cell lines. ARPE-19 is a well-characterized spontaneously immortalized RPE culture established in 1996^90^. Regardless of the chosen cell line, our data support better consideration of ECM properties to improve cell culture experiments and achieve results with higher comparability to and relevance for *in vivo* conditions.

In summary, our data indicate a significant impact of substrate stiffness on the transcriptome of RPE. Stiffness-dependent miRNA expression changes barely relate to differentially expressed mRNAs and are more likely involved in tissue homeostasis. Wnt/beta-catenin signaling is a target of miRNAs more highly expressed on stiffer substrates. Associated targets like MITF with a known impact on RPE and the endoribonuclease Dicer show a stiffness-dependent expression pattern. The findings underline the need for a more comprehensive representation of ECM properties in cell culture experiments. Further validation using primary RPE would be useful to assess the impact of the described changes on the pathogenesis and potential therapeutic approaches of degenerative retinal diseases.

## Methods

### Cell culture

Cells from the human RPE cell line ARPE-19^90^ were purchased from the American Type Culture Collection (ATCC CRL2302 ARPE-19 Retinal Pigment Epithelium Human, Lot Number 63478793). DMEM/Ham’s F-12 media, 10% FBS, L-Glutamine (${200}\,\hbox {mM}$) and Penicillin-Streptomycin (10,00${0}\,\hbox {U/ml}$ Penicillin, ${10}\,\hbox {mg/ml}$ Streptomycin) were obtained from Biochrom (Cambridge, United Kingdom). MEM Non-Essential Amino Acids Solution (100x) and Insulin-Transferrin-Selenium (100x) were acquired from Life Technologies (Thermo Fisher Scientific, Waltham, MA, USA). HEPES buffer (1M) was obtained from PAN-Biotech (Aidenbach, Germany). Standard conditions for cell culture were applied (37${}^{\circ }\textrm{C}$ temperature, 5% CO_2_ supplement, 90% relative humidity). Media was changed twice a week. Only early passages of cells (P<10) in a confluent state were used for conducting experiments.

### Flexible substrates

For examination of cellular responses to mechanical properties of the adhesion substrate, PA gels of different substrate stiffness were casted^10,91^ and coated with fibronectin (10 $\upmu$g/ml PBS) as previously reported^19^. 30% of Acrylamide, Rotiphorese B with 2% of Bis-Acrylamide, (3-Acrylamidopropyl)trimethylammonium chloride solution, Ammonium peroxydisulfate for 10% Ammonium peroxydisulfate and TEMED were obtained from Carl Roth (Karlsruhe, Germany). PBS was purchased from Gibco (Thermo Fisher Scientific, Waltham, MA, USA). Nuclease-Free Water was acquired from Sigma-Aldrich (Merck, Darmstadt, Germany). Substrate stiffness was determined by the proportion of Rotiphorese B and systematically controlled by the process of bio-indentation (Bioindenter UNHT3 Bio, Anton Paar, Ostfildern-Scharnhausen, Germany)^92–94^. For conducting stiffness experiments, PA gels of ${30}\,\hbox {kPa}$ ($\hat{=}$ 0,3% of Rotiphorese B) and ${80}\,\hbox {kPa}$ ($\hat{=}$ 0,8% of Rotiphorese B) as well as TCP were coated with Human Plasma Fibronectin Purified Protein (Sigma-Aldrich, Merck, Darmstadt, Germany) and used as adhesion substrates. To facilitate cell growth on soft tissues, adapted cell numbers were used as follows: 1,200,000 cells on PA gels of ${30}\,\hbox {kPa}$, 800,000 on PA gels of ${80}\,\hbox {kPa}$ and 500,000 on TCP.

### RNA isolation and protein isolation

RNAs and proteins were isolated after three weeks of confluent cultivation. ${35}\,\hbox {mm}$ plates were used for experiments and a defined number of ${35}\,\hbox {mm}$ plates were pooled according to the amount of cellular material needed for further analysis. Normalized amounts of material were used for cDNA synthesis and western blotting analysis. miRNeasy and Qiagen RNeasy (Qiagen, Venlo, The Netherlands) were used for isolating mRNAs and sRNAs. RNA sample quality was assessed by a lab-on-a-chip system (Bioanalyzer, Agilent, Santa Clara, CA, USA), total amount of RNA was assessed by a spectrophotometry-based assay (NanoDrop ND-1000 Spectrophotometer, Peqlab Biotechnologie, VWR, Radnor, PA, USA). Total sRNA content was determined with a fluorescence-based assay (Quant-iT, Invitrogen, Thermo Fisher Scientific, Waltham, MA, USA). Lysis buffer was used for isolating proteins (TRIS [${100}\,\hbox {mM}$], Sigma-Aldrich, Merck, Darmstadt, Germany; EDTA [${500}\,\hbox {mM}$], Serva Electrophoresis, Heidelberg, Germany; Triton X-100, Sigma-Aldrich, Merck, Darmstadt, Germany; sterile water, Fresenius Kabi, Bad Homburg, Germany). Total protein content was determined by bicinchoninic acid (BCA) assay (Pierce BCA Protein Assay Kit, Thermo Scientific, Thermo Fisher Scientific, Waltham, MA, USA).

### Next generation sequencing and qPCR

In a pilot experiment of $n={1}$, sRNAs and mRNAs were studied by NGS (in cooperation with GenXPro, Frankfurt, Germany). Differential expression analysis was performed using *NOISeq*^95,96^. RT^2^ Profiler PCR Array Human WNT Signaling Pathway (Qiagen, Venlo, The Netherlands), conducted as a pilot project with $n={1}$, was used to study RNA expression of the Wnt/beta-catenin pathway. Extensive probe-based qPCR (TaqMan, Thermo Fisher Scientific, Waltham, MA, USA) using the $2^{-\Delta \Delta C_{\text {T}}}$ method^97^ was performed to verify preliminary data. Data was analyzed using *rstatix*^98^ and visualized using *ggpubr*^99^.

### Gene ontology pathway analysis

Predicted targets of differentially expressed miRNAs were calculated based on *Targetscan*^100^ using *isomiRs*^101^. Functional analysis was performed based on the *GO*^102,103^ using *clusterProfiler*^104^. Data was further validated by qPCR and western blotting.

### Western blotting analysis

The Mini-Protean wet electroblotting system by Bio-Rad Laboratories (Hercules, CA, USA) was used for performing western blotting. Protein bands were marked by horseradish peroxidase conjugated antibodies and detected with the help of Amersham ECL Western Blotting Detection Reagents (RPN2109, GE Healthcare, Chicago, IL, USA) and Pierce ECL Plus Western Blotting Substrate (32134, Thermo Scientific, Thermo Fisher Scientific, Waltham, MA, USA). Each individual plot was generated from a western blot analysis that was performed using a single membrane.

### Statistics and data visualization

Data analysis was performed using *R 3.5.2*^105^ in *RStudio 1.1.463*^106^ with the included *base* package as well as the packages from the *tidyverse* collection^107^, especially *dplyr*^108^ for data manipulation and *ggplot2*^109^ for data visualization. Particularly used *R* packages are mentioned respectively. Image processing was performed using *ImageJ 1.52u*^110^, *Inkscape 1.0.1*^111^ and *GIMP 2.10.18*^112^.

### Supplementary Information

Supplementary Information.

## Data Availability

The datasets generated and analyzed during the current study are available in the Gene Expression Omnibus (GEO) repository, https://www.ncbi.nlm.nih.gov/geo/query/acc.cgi?acc=GSE225642.
