# Supplementary material for: The impact of substrate stiffness on morphological, transcriptional and functional aspects in RPE
Source: Sci Rep. 2024 Mar 29;14:7488. doi: 10.1038/s41598-024-56661-7 (PMC11344127; doi:10.1038/s41598-024-56661-7)
Supplement: Supplementary file 1 — Supplementary Information. [file 41598_2024_56661_MOESM1_ESM.pdf]

Gene	$\log_2 FC$	Gene	$\log_2 FC$	Gene	$\log_2 FC$
WIF1	-1,814	*CCND2*	+3,376	*FZD2*	+1,536
FGF4	-1,664	*CXXC4*	+2,976	*APC*	+1,366
PITX2	-1,174	*SFRP4*	+2,826	*KREMEN1*	+1,236
WNT6	-1,054	*WNT2B*	+2,096	*FZD8*	+1,186
DVL1	-1,014	*SFRP1*	+1,926	*FZD4*	+1,086
		DKK3	+1,766	*PRICKLE1*	+1,066

Table S1. Stiffness-dependent mRNA expression profile of members of the Wnt/beta-catenin signaling pathway in ARPE-19 cells (RT^2 Profiler PCR Array). Declaration of differences in expression between soft ($E = 30\,\mathrm{kPa}$) and stiff ($E = 80\,\mathrm{kPa}$) substrate as $\log_2 FC$. Cutoff of genes shown based on $|\log_2 FC| \geq 1$. Resulting selection of 17 genes with a change in expression out of 84 investigated genes. Order based on $\log_2 FC$. Normalization on *ACTB*, *B2M*, *GAPDH*, *HPRT1* and *RPLP0* as endogenous controls. Expression data from $n = 1$ experiment.

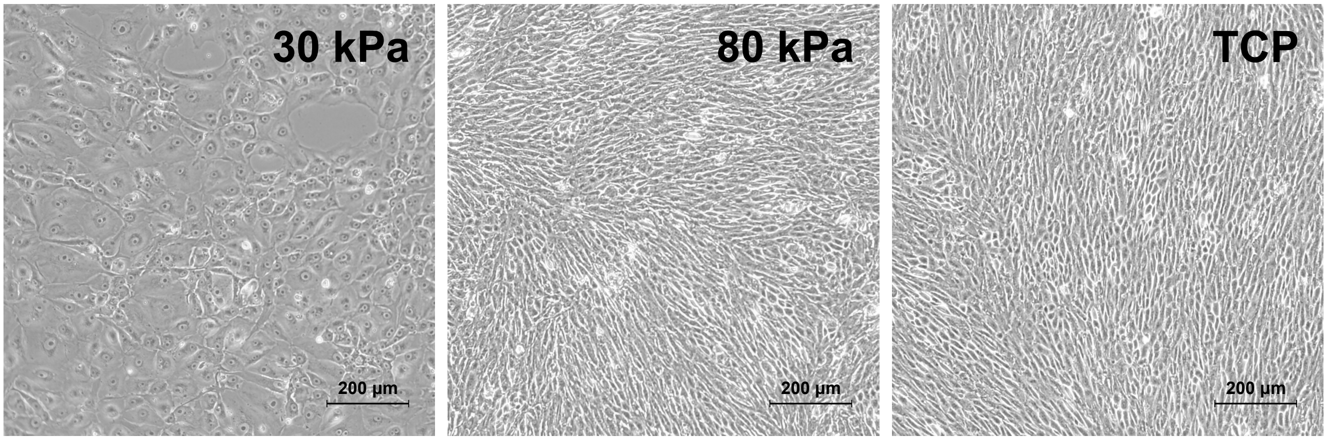

Figure S1. Stiffness-dependent morphology of ARPE-19 cells after three weeks of culture on substrates of different Young's modulus (phase contrast microscopy). Morphology of cells on soft ($E = 30\,\mathrm{kPa}$) and stiff ($E = 80\,\mathrm{kPa}$) substrate as well as on standard tissue culture plastic (TCP) is shown.

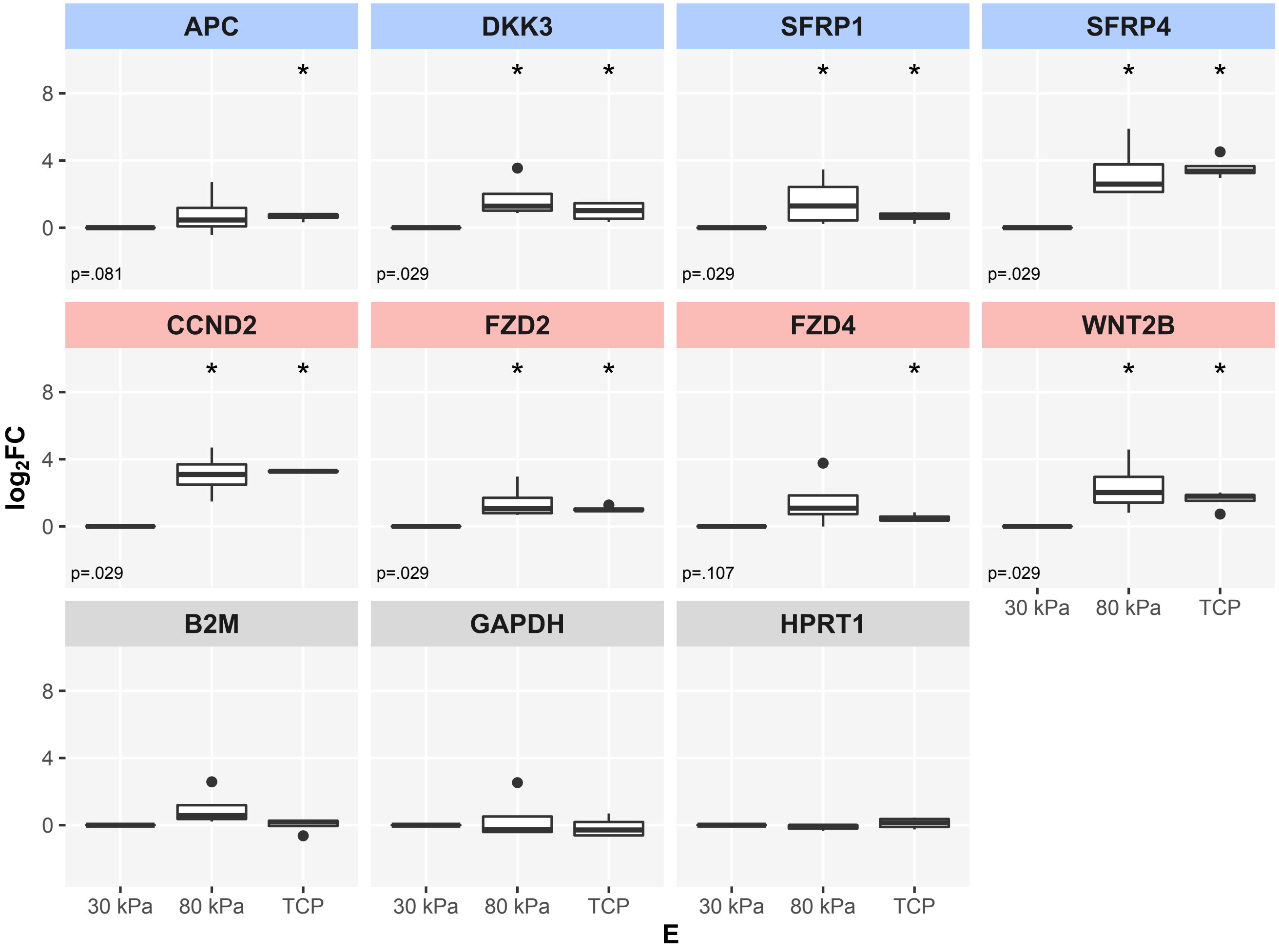

Figure S2. Stiffness-dependent transcription of members of the Wnt/beta-catenin signaling pathway in ARPE-19 cells for validation of qPCR array data (qPCR). Expression data from $n = 4$ independent experiments, visualization as $\log_2 FC$ with $E = 30\,\text{kPa}$ as reference. Coloring of antagonistic (blue) and agonistic (red) impact on the activity of the Wnt/beta-catenin signaling pathway. Normalization on *B2M*, *GAPDH* and *HPRT1* as endogenous controls (gray). Statistical significance between each two interventions was assessed by Wilcoxon-Mann-Whitney test with $E = 30\,\text{kPa}$ as reference. $p \leq 0,05$ (*), $p \leq 0,01$ (**) and $p \leq 0,001$ (***). Global p-values were determined using Kruskal-Wallis test. Benjamini-Hochberg procedure was applied for multiple testing correction.

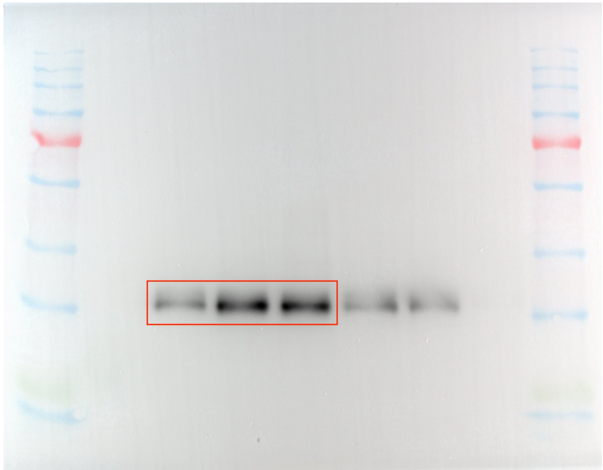

Figure S3. Stiffness-dependent expression of SFRP1 ($\approx 35\,\text{kDa}$) in ARPE-19 cells (full western blot) with the three lanes of interest from left to right representing $E = 30\,\text{kPa}$, $E = 80\,\text{kPa}$ and TCP (red rectangle).

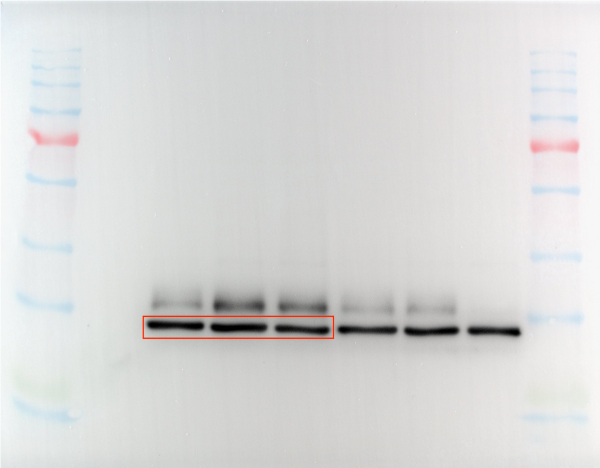

Figure S4. Loading control GAPDH ($\approx 38\,\text{kDa}$) in ARPE-19 cells (full western blot) with the three lanes of interest from left to right representing $E = 30\,\text{kPa}$, $E = 80\,\text{kPa}$ and TCP (red rectangle).

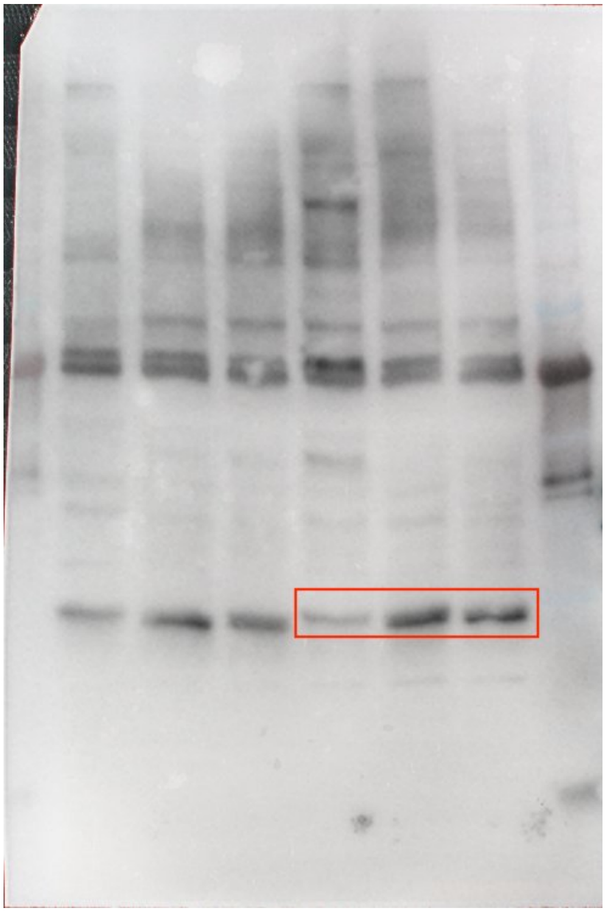

Figure S5. Stiffness-dependent expression of Wnt-2b ($\approx 41\,\text{kDa}$) in ARPE-19 cells (full western blot) with the three lanes of interest from left to right representing $E = 30\,\text{kPa}$, $E = 80\,\text{kPa}$ and TCP (red rectangle).

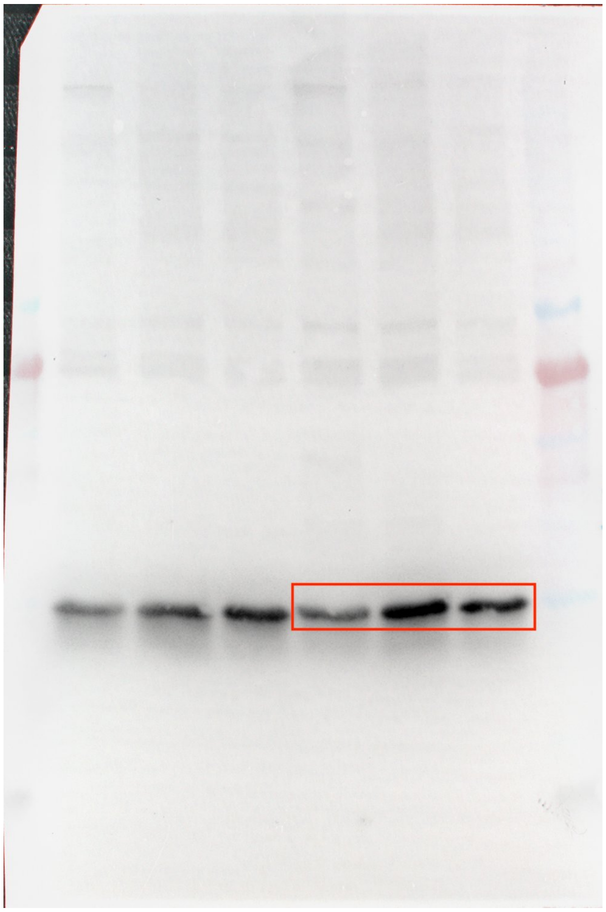

Figure S6. Loading control GAPDH ($\approx 38\,\text{kDa}$) in ARPE-19 cells (full western blot) with the three lanes of interest from left to right representing $E = 30\,\text{kPa}$, $E = 80\,\text{kPa}$ and TCP (red rectangle).

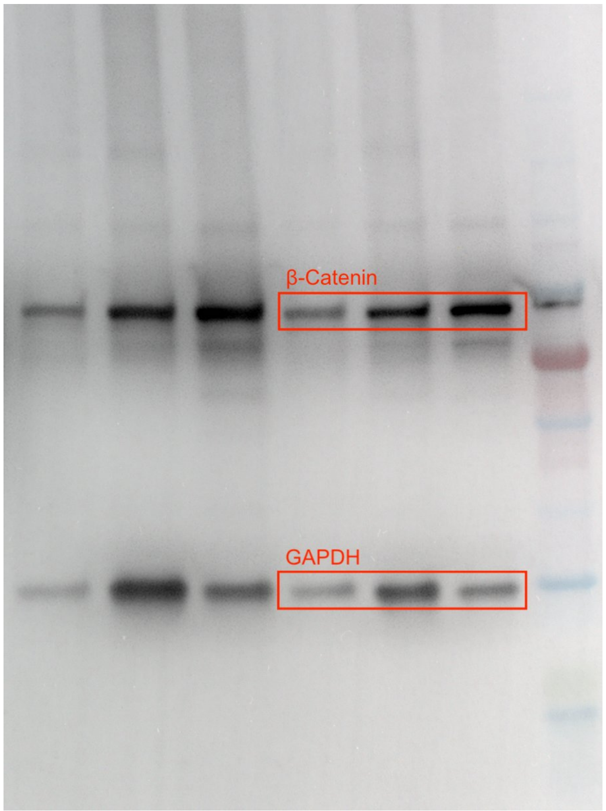

Figure S7. Stiffness-dependent expression of beta-catenin ($\approx 92\,$kDa) and loading control GAPDH ($\approx 38\,$kDa) in ARPE-19 cells (full western blot) with the three lanes of interest from left to right representing $E = 30\,$kPa, $E = 80\,$kPa and TCP (red rectangles).

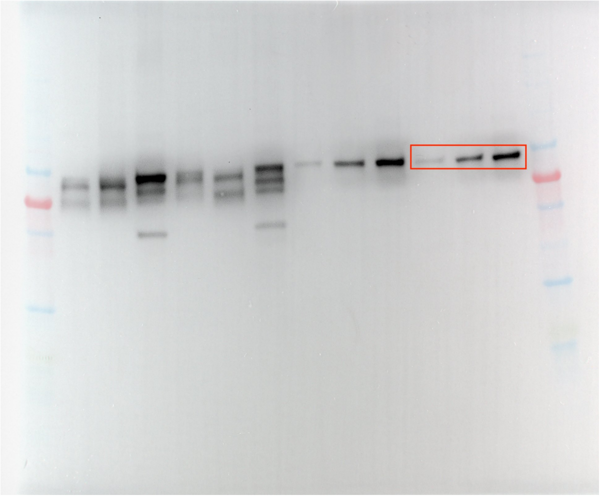

Figure S8. Stiffness-dependent expression of Non-phospho (active) beta-catenin (Ser45) ($\approx 92\,$kDa) in ARPE-19 cells (full western blot) with the three lanes of interest from left to right representing $E = 30\,$kPa, $E = 80\,$kPa and TCP (red rectangle).

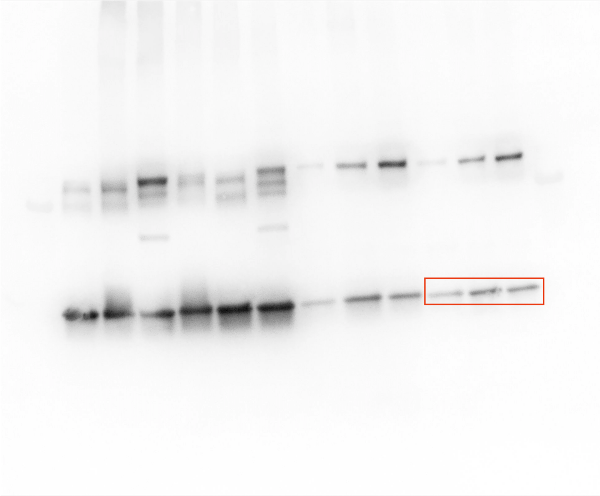

Figure S9. Loading control GAPDH ($\approx 38\,$kDa) in ARPE-19 cells (full western blot) with the three lanes of interest from left to right representing $E = 30\,$kPa, $E = 80\,$kPa and TCP (red rectangle).

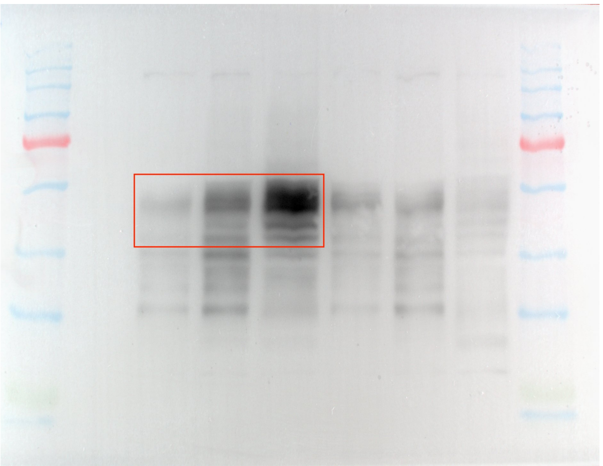

Figure S10. Stiffness-dependent expression of MITF (≈ 50 kDa to 75 kDa) in ARPE-19 cells (full western blot) with the three lanes of interest from left to right representing $E = 30\,\text{kPa}$, $E = 80\,\text{kPa}$ and TCP (red rectangle).

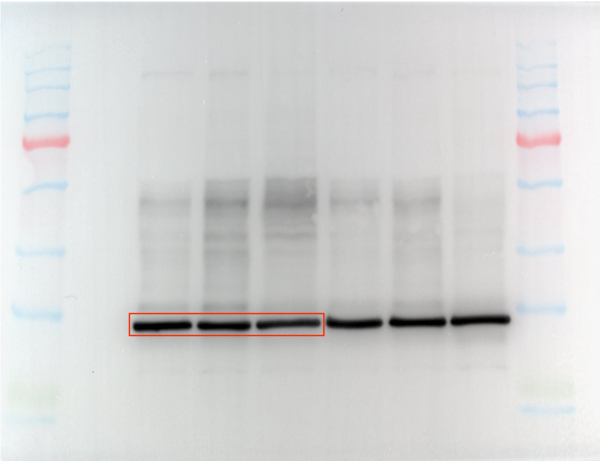

Figure S11. Loading control GAPDH (≈ 38 kDa) in ARPE-19 cells (full western blot) with the three lanes of interest from left to right representing $E = 30\,\text{kPa}$, $E = 80\,\text{kPa}$ and TCP (red rectangle).

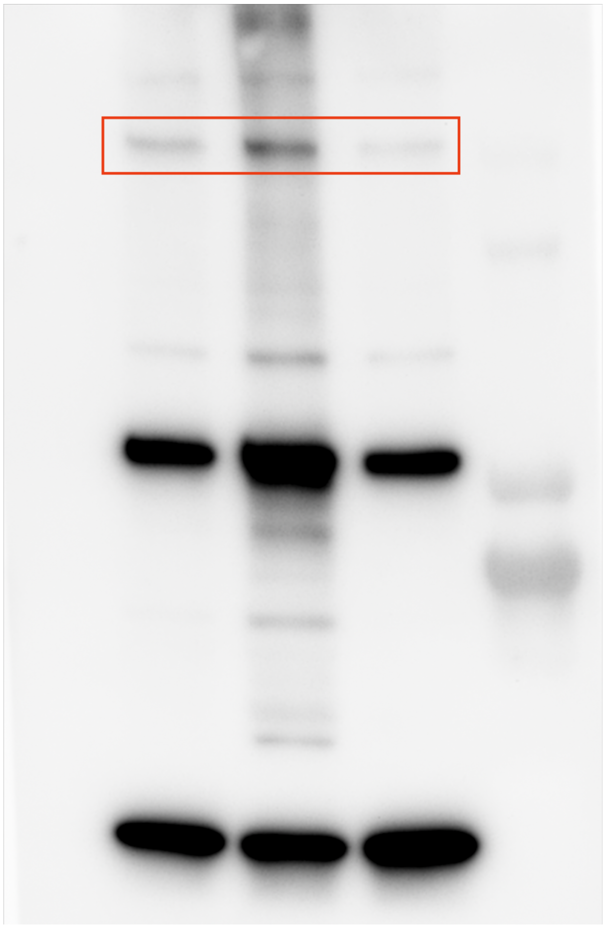

Figure S12. Stiffness-dependent expression of Dicer (≈ 220 kDa) in ARPE-19 cells (full western blot) with the three lanes of interest from left to right representing $E = 30\,\text{kPa}$, $E = 80\,\text{kPa}$ and TCP (red rectangle).

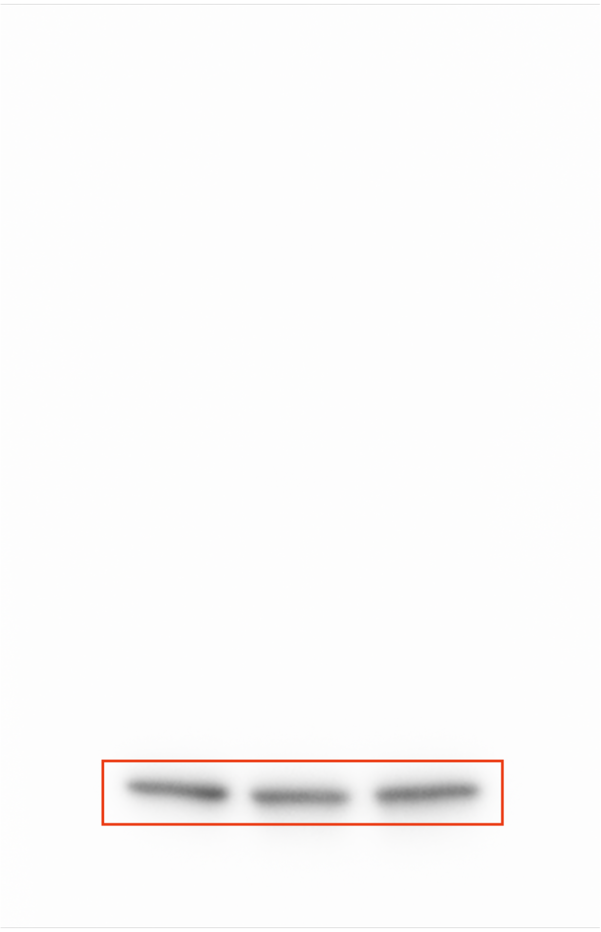

Figure S13. Loading control GAPDH (≈ 38 kDa) in ARPE-19 cells (full western blot) with the three lanes of interest from left to right representing $E = 30\,\text{kPa}$, $E = 80\,\text{kPa}$ and TCP (red rectangle).
